# Cannabidiol does not attenuate acute delta‐9‐tetrahydrocannabinol‐induced attentional bias in healthy volunteers: A randomised, double‐blind, cross‐over study

**DOI:** 10.1111/add.16353

**Published:** 2023-10-11

**Authors:** Dominic Oliver, Amir Englund, Edward Chesney, Lucy Chester, Jack Wilson, Simina Sovi, Stina Wigroth, John Hodsoll, John Strang, Robin M. Murray, Tom P. Freeman, Paolo Fusar‐Poli, Philip McGuire

**Affiliations:** ^1^ Department of Psychiatry University of Oxford Oxford UK; ^2^ NIHR Oxford Health Biomedical Research Centre Oxford UK; ^3^ Department of Psychosis Studies, Institute of Psychiatry, Psychology and Neuroscience King's College London London UK; ^4^ Addictions Department, Institute of Psychiatry, Psychology and Neuroscience King's College London London UK; ^5^ Laboratoire Didier Jutras‐Aswad Centre de Recherche du Centre hospitalier de l'Université de Montréal Montréal QC Canada; ^6^ The Matilda Centre for Research in Mental Health and Substance Use, Level 6, Jane Foss Russell Building, G02 The University of Sydney NSW Australia; ^7^ Department of Biostatistics and Health Informatics, Institute of Psychiatry, Psychology and Neuroscience King's College London London UK; ^8^ South London and the Maudsley National Health Service Foundation Trust London UK; ^9^ Department of Psychology University of Bath Bath UK; ^10^ Department of Brain and Behavioural Sciences University of Pavia Pavia Italy; ^11^ Department of Psychiatry and Psychotherapy Ludwig‐Maximilian‐University Munich Munich Germany

**Keywords:** attentional bias, cannabidiol, cannabis, cannabis use disorder, CBD, delta‐9‐tetrahydrocannabinol, THC

## Abstract

**Aims:**

To test how attentional bias and explicit liking are influenced by delta‐9‐tetrahydrocannabinol (THC) and whether these effects are moderated by cannabidiol (CBD).

**Design:**

Double‐blind, randomised, within‐subjects cross‐over study.

**Setting:**

NIHR Wellcome Trust Clinical Research Facility at King's College Hospital, London, United Kingdom.

**Participants/Cases:**

Forty‐six infrequent cannabis users (cannabis use <1 per week).

**Intervention(s):**

Across four sessions, participants inhaled vaporised cannabis containing 10 mg of THC and either 0 mg (0:1 CBD:THC), 10 mg (1:1), 20 mg (2:1) or 30 mg (3:1) of CBD, administered in a randomised order and counter‐balanced across participants (a total of 24 order groups).

**Measurements:**

Participants completed two tasks: (1) Attentional Bias (AB), comparing reaction times toward visual probes presented behind 28 target stimuli (cannabis/food) compared with probes behind corresponding non‐target (neutral) stimuli. Participants responding more quickly to probes behind target than non‐target stimuli would indicate greater attentional bias to cannabis/food; (2) Picture Rating (PR), where all AB stimuli were rated on a 7‐point pleasantness scale, measuring explicit liking.

**Findings:**

During the AB task, participants were more biased toward cannabis stimuli in the 0:1 condition compared with baseline (mean difference = 12.2, 95% confidence intervals [CIs] = 1.20–23.3, *d* = 0.41, *P* = 0.03). No other significant AB or PR differences were found between cannabis and food stimuli between baseline and 0:1 condition (*P* > 0.05). No significant CBD effect was found on AB or PR task performance at any dose (*P* > 0.05). There was additionally no cumulative effect of THC exposure on AB or PR outcomes (*P* > 0.05).

**Conclusions:**

A double‐blind, randomised, cross‐over study among infrequent cannabis users found that inhaled delta‐9‐tetrahydrocannabinol increased attentional bias toward cannabis in the absence of explicit liking, a marker of liability toward cannabis use disorder. At the concentrations normally found in legal and illegal cannabis, cannabidiol had no influence on this effect.

## INTRODUCTION

Cannabis containing high concentrations of delta‐9‐tetrahydrocannabinol (THC) has been shown to increase the risk of developing symptoms of problematic cannabis use [1] and the onset and severity of cannabis use disorder (CUD) [2, 3, 4, 5]. THC concentrations in cannabis have been steadily increasing over time [6], and although people partially titrate their THC consumption according to the THC concentration of their cannabis, they often do not fully compensate [7]. This means that increases in THC concentrations in street purchases of cannabis will result in cannabis users receiving higher doses of THC that may increase the risk of poorer outcomes.

Following exposure to the rewarding effects of an addictive substance, users theoretically become hyper‐sensitised to drug cues in their environment leading to attributing abnormally high incentive salience to these cues [8]. This heightened attribution of salience can result in automatic biasing of attention toward drug stimuli. These cognitive processes are the basis of attentional bias and picture rating tasks, which model implicit wanting and explicit liking, respectively. The concept of attentional bias is a key mechanism within the development and maintenance of substance use disorders, giving the importance of drug stimuli and perceived reward in drug craving [9]. There is consistent evidence of greater bias toward cannabis stimuli in regular cannabis users [10] and people with CUD [11] compared to healthy controls. It has, therefore, been considered as a biomarker of addiction liability or target for clinical intervention in substance use disorders [12], but the evidence for their effectiveness has been mixed [13].

One intervention for CUD that has attracted interest is cannabidiol (CBD), the second most common cannabinoid in cannabis, notably non‐intoxicating with a benign side effect profile [14]. A case study following a patient with cannabis dependence identified a significant decrease in withdrawal symptoms after 6 days of CBD administration [15]. This has since been reinforced by a randomised double‐blind placebo‐controlled trial showing that 400 mg and 800 mg of oral CBD can reduce cannabis use in individuals with CUD [16]. In terms of attentional bias toward cannabis stimuli, this has been explored previously in a naturalistic study by Morgan *et al*. [17]. Regular cannabis users donated their own cannabis for analysis and participants were stratified according to the CBD:THC ratio of their cannabis, either low (~1:100) or high (~1:3). The participants then completed tasks indexing attentional bias and explicit liking of cannabis stimuli both sober and when intoxicated with their own cannabis. Attentional bias and explicit liking of cannabis stimuli were maintained in regular users when intoxicated, but this effect was less pronounced with administration of cannabis containing higher CBD:THC ratios [17].

The effect of acute THC administration on attentional bias in infrequent cannabis users is under‐researched. As in Morgan *et al*. [17], co‐administration of CBD may mitigate against increases in bias toward cannabis stimuli. Reducing bias at this early stage could provide preliminary support for controlling the CBD content of cannabis to reduce the incidence of CUD among people who use cannabis infrequently and could offer a potential strategy for universal harm reduction [18]. There has yet to be a controlled study testing a range of CBD:THC ratios relevant for people who use cannabis on attentional bias and explicit liking of cannabis. This study aimed to compare the acute effects of cannabis administration containing four different CBD:THC ratios (0:1, 1:1, 2:1 and 3:1) on attentional bias and explicit liking of cannabis and food stimuli in infrequent cannabis users. We hypothesised that THC administration would result in higher attentional bias to cannabis and food stimuli and that this effect will decrease as the CBD:THC ratio is increased.

## METHODS

The study was approved by the King's College London Research Ethics Committee (RESCMR‐16/17‐4163). All participants provided written informed consent and the study was conducted in compliance with the principles of Good Clinical Practice, the Declaration of Helsinki (1996). The study was registered on Open Science Framework (https://osf.io/kt3f7) and clinicaltrials.gov (NCT05170217). Expanded details such as randomisation, blinding and sample size calculation of the study can be seen in Englund *et al*. [19].

### Design

This randomised, double‐blind, four‐arm, within‐subjects study was conducted at the National Institute for Health Research (NIHR) Wellcome Trust Clinical Research Facility (CRF) at King's College Hospital, London, United Kingdom. The study design was a four‐phase cross‐over with each phase corresponding to one of the four CBD:THC dosing ratios: 0:1, 1:1, 2:1, 3:1. Each participant was required to complete all phases corresponding to all ratios of cannabis preparation. For four conditions there are 24 possible permutations of sequential order, for example, Visit 1, 2:1–Visit 2, 0:1–Visit 3, 1:1–Visit 4, 3:1. Randomised sequences were generated in blocks with the first 24 participants allocated each of the 24 possible order of ratios, as were the next 24 and so on. Where there were fewer participants than possible order sequences, each participant received a random selection from the 24, sampled without replacement. The randomisation list was generated by a statistician not involved with the study, using a customised randomisation script generated in R software v 3.1 (available on request).

The randomisation was double blinded to both researchers and participants. The randomisation list was passed from the independent statistician to the Maudsley Pharmacy who prepared the cannabis preparations. The pharmacy dispensed the study drug to a blinded researcher. The cannabis preparation was then loaded into the filling chamber and vaporised by a research nurse who was not involved with any other study procedures. On completion of data collection and entry the randomisation schedule was revealed to the research team before data analysis. Participants attended a baseline session, followed by four experimental visits, with a minimum 1‐week wash‐out period between each experimental visit (median duration between experiments was 14 days; interquartile range = 20).

### Participants

Forty‐six healthy volunteers were recruited by email advertisements sent to staff and students at King's College London. Inclusion criteria were: age 21 to 50 years, had used cannabis at least once in the past, able and willing to provide written informed consent, willing to give blood samples and be fluent in English. Exclusion criteria were: past or present a major mental, physical illness or substance use disorder, current use of psychotropic medication, history of antipsychotic or antidepressant medication use, first degree relative with a psychotic illness, positive urine drug screen, past 24‐hour use of alcohol or tobacco, being pregnant/planning pregnancy or lactating, mean cannabis use >1/week over the last 12 months, past use of synthetic cannabinoids, score of 5 or more on the Fagerstrom Nicotine Dependence Questionnaire, body mass index <18 or >30, having participated in a drug study within the past 30 days or a research study during the course of the current study and a known sensitivity or allergy to cannabis or lorazepam.

### Procedure

At baseline, participants were assessed for study eligibility and practiced the inhalation procedure before administration of the cognitive tasks. Written informed consent was provided by all participants before collection of any data. At baseline and all experimental visits, urine drug and pregnancy screen as well as alcohol and carbon monoxide breath tests (<10 p.p.m. CO to verify 12 hour tobacco abstinence) were completed. Participants were asked to avoid using cannabis and all other illicit drugs during the entire course of the study, including the periods between sessions.

Before each experimental visit participants had their usual breakfast and amount of caffeine—caffeine was not allowed again until completion of cognitive tests. An intravenous cannula was inserted before participants were administered vaporised cannabis (detailed below). Fifteen minutes after completion of inhalation, participants completed the cognitive tasks. Participants were discharged after a field sobriety test, having been informed of safety protocols and provided with a 24‐hour emergency number.

### Study drug and administration

The study drug was provided in the form of granulated cannabis inflorescence by Bedrocan BV produced in accordance with Good Manufacturing Practice and conforms to the European Medicines Agency's contaminant levels for products used in the respiratory tract. Each cannabis dose consisted of 10 mg of THC (two standard THC units) [20] and either 0, 10, 20 or 30 mg of CBD. Participants were given preparations with CBD:THC ratios of 0:1, 1:1, 2:1 and 3:1, in a random order across visits. Bedrocan (22.6% THC, 0.1% CBD), Bedrolite (7.5% CBD, 0.3% CBD) and Bedrocan placebo (<0.01% THC) were used to provide cannabis containing THC, CBD and placebo, respectively. The placebo cannabis was added to ensure that all preparations had the same weight (see Table S1).

Cannabis preparations were administered using a Volcano Medic Vaporizer (Storz‐Bickel GmbH) [21, 22]. Each preparation was vaporised at 210°C into a transparent polythene bag. Once filled, this was encased with an opaque bag to ensure blinding (a higher CBD:THC ratio produces a denser vapour). Inhalation was standardised by asking participants to hold their breath for 8 seconds before exhaling, with an 8‐second break between inhalations. The procedure continued until the contents of two bags had been completed.

### Blood collection and analysis

Venous blood samples were taken before drug administration, and at 0, 5, 15 and 90 minutes following the final exhalation, alongside blood pressure, heart rate and temperature. The concentration of Δ9‐THC, 11‐OH‐Δ9THC (OH‐THC), 11‐COOH‐Δ9‐THC (COOH‐THC), CBD and 7‐OH‐CBD were determined using high performance liquid chromatography mass spectrometer at the Mass Spectrometry Facility, King's College London [23].

### Attentional bias task

**FIGURE 1:**
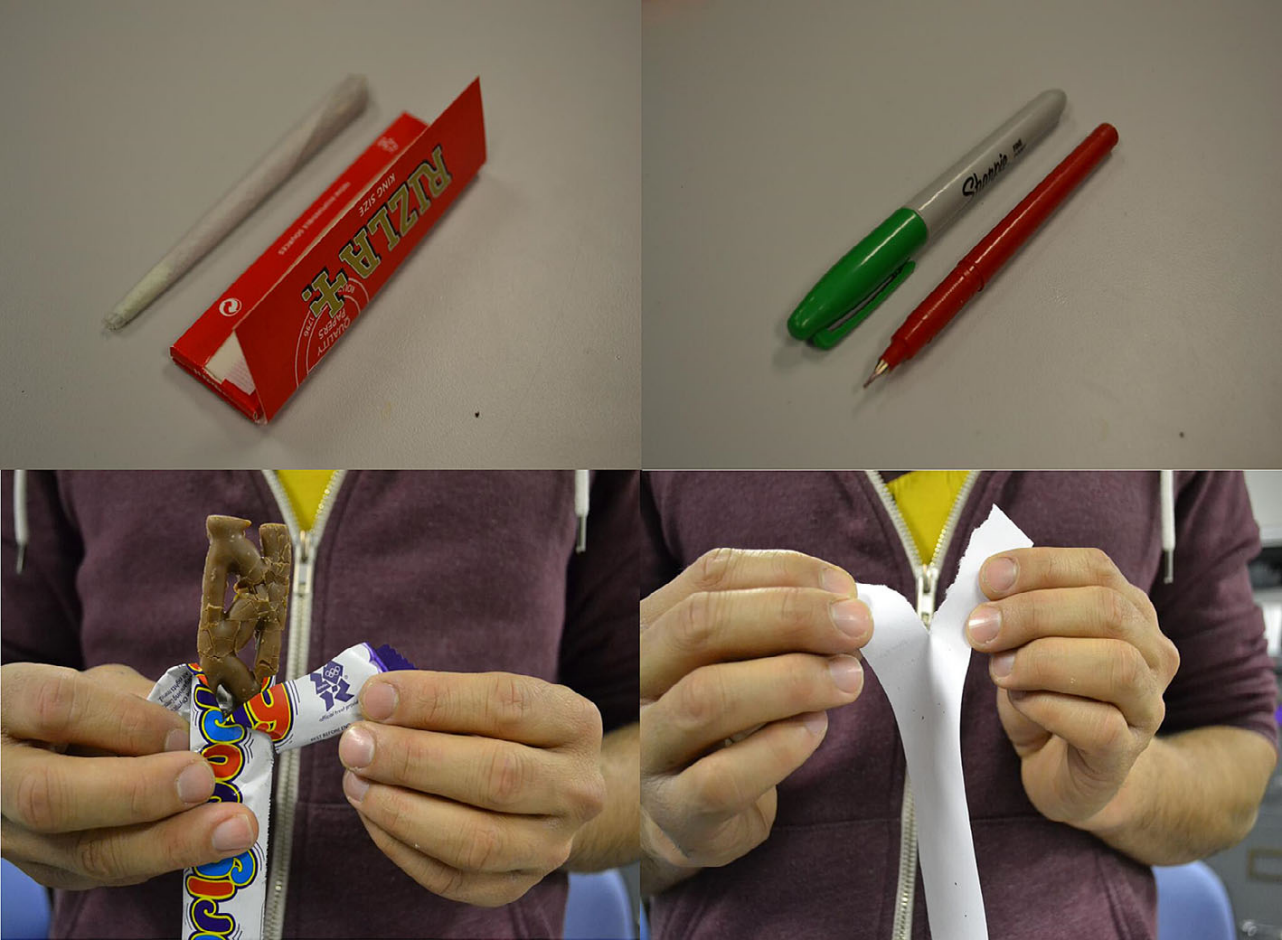
Examples of cannabis (top left)/neutral (top right) and food (bottom left)/neutral (bottom right)‐matched pairs of images.

A computer‐based task presenting target (cannabis/food) and non‐target (neutral) stimuli was used to assess attentional bias of both cannabis and food stimuli. These stimuli were presented in pairs, consisting of a target image and a neutral image that closely matched the target's structure (see Figure 1).

The task itself consisted of four practice trials and 112 experimental trials featuring seven cannabis and seven food stimuli pairs (each presented with a matching neutral image). Different image pairs were used on each visit to control for order effects. Over five visits, participants were presented a total of 35 colour images of cannabis stimuli with 35 respective neutral stimuli and 35 colour images of food stimuli with 35 respective neutral stimuli. Each trial began by showing a central fixation cross for 1000 ms, after which two images appeared (one target, the other non‐target) for a short (250 ms) or long (500 ms) duration. Next, both images disappeared, and an arrow flashed behind one of the two images. A congruent trial occurred when the arrow flashed behind the cannabis/food image, whereas an incongruent trial occurred when the arrow flashed behind the neutral image. Participants had to respond, as accurately and quickly as possible, by pressing the key indicating the direction in which the arrow was pointing (upward or downward). Stimulus presentation duration and position were counterbalanced across all trials.

Trials with reaction times <100 ms, >1000 ms or with incorrect responses were excluded from the initial analysis in line with earlier studies [17]. Attentional bias was calculated as the difference in reaction time taken to respond to the arrow when it flashed behind the non‐target, as opposed to target (RTneutral−RTcannabis/food). More positive values indicated stronger biases toward the cannabis/food stimuli compared to the neutral stimuli.

### Picture‐rating task

Following the attentional bias task, participants were presented with the same cannabis and food stimuli from the attentional bias task in a random order. Participants were asked to rate the pleasantness of the images on a 7‐point scale, ranging from −3 (very unpleasant) to +3 (very pleasant).

### Statistical analysis

A per‐protocol analysis was used as 89% of dropouts occurred after cannabis inhalation, but before data collection (see Results). The study was originally designed for *n* = 45 participants (based on power calculation of primary outcome in main study [19]), which would be powered to detect an effect size of *d* = 0.43 at 80% power and α = 0.05. Outliers were identified using Rosner's test and removed. Multiple imputation chain equations (MICE) were used to impute missing values among the 46 participants included in the analysis using the MICE package (version 3.13.0) [24], following no detection of deviation from missing completely at random (MCAR) based on Little's MCAR test [25]. Little's MCAR test was run on a dataset including visit order, CBD:THC ratio, cannabis bias and food bias. There were no missing data for picture rating scores, so these were not included. To test the impact of imputation, analyses were repeated without imputed values.

The effect of THC was determined by comparing outcome scores from the baseline visit with those following administration with THC alone (0:1) using paired *t* tests. We used linear mixed models to assess the effect of varying the CBD:THC ratio on cognitive task performance. The four CBD:THC ratios (0:1, 1:1, 2:1, 3:1) were included as a fixed effect, with participant as a random effect to account for the dependency between repeated measures. All six contrasts were of interest (0:1 vs 3:1, 0:1 vs 2:1, 0:1 vs 1:1, 1:1 vs 3:1, 2:1 vs 1:1). α Was set as *P* < 0.05. *P* values for linear mixed models were corrected using the Tukey method.

The following sensitivity analyses were conducted for attentional bias: (i) stratifying by stimulus presentation duration to investigate potentially greater magnitude of attentional bias change in shorter stimulus presentation as these will theoretically involve less conscious control; (ii) stratifying by congruent/incongruent trials; (iii) including trials previously excluded because of duration or incorrect responses.

Potential cumulative effects of THC on attentional bias and picture rating were investigated by replacing CBD:THC ratio with visit number in the model. The number of days since the previous experiment was subsequently entered into the model as a covariate as a sensitivity analysis.

Relationships between both peak and area under the curve (AUC) plasma THC/CBD and attentional bias/picture rating scores were tested using Pearson's correlations. For the AUC analyses, values were baseline corrected before using the spline method using the bayestestR package (version 0.7.5.1) [26].

All analyses were conducted using R version 4.1.0. lme4 (version 1.1‐26) [27] was used to fit the linear mixed effects models and estimated marginal mean (EMM) contrasts were calculated using the emmeans package (version 1.5.2‐1) [28].

## RESULTS

### Participants and demographics

Eighty potential participants were screened from which 64 were randomised and 46 completed the study between November 2017 and June 2019 (see CONSORT flow diagram Figure S8). Of the 18 randomised participants who were later excluded (one was excluded at completion, two were following the second visit and the remaining did not complete their first visit), 12 dropped out because of unpleasant drug effects, one because of a positive drug screen, one because of an absence of subjective and objective THC effects and four for reasons unrelated to study procedures. Demographics for included and excluded participants (excluding participant who lacked THC response) are presented in Table 1. One baseline dataset had incomplete data on all attentional bias and picture rating outcomes and was excluded from the final analysis: 229/230 (99.6%) datasets were included in the final analysis. Further to this, *n* = 13 (0.1%) data points were excluded as outliers and imputed using MICE.

**TABLE 1:** Sociodemographics.

	Completers (*n* = 46)	Drop‐outs (*n* = 17)
Sex; *n* (%)		
Male	25 (54.3)	6 (35.3)
Female	21 (45.7)	11 (64.7)
Age; mean (SD)	26.62 (4.94)	25.88 (4.41)
Ethnicity; *n* (%)		
White	31 (67.4)	12 (70.6)
Asian	11 (23.9)	1 (5.9)
Mixed	3 (6.5)	4 (23.5)
Black	1 (2.2)	0 (0)
Education; *n* (%)		
A levels	9 (19.6)	2 (11.8)
Vocational	0 (0)	1 (5.9)
University/professional qualification (degree+)	18 (39.1)	11 (64.7)
Postgraduate degree	19 (41.3)	3 (17.6)
Weight (kg); mean (SD)	70.68 (11.3)	66.14 (1.97)
BMI (kg/m^2^); mean (SD)	23.72 (2.57)	22.62 (1.97)
Body fat (%), male; mean (SD)	15.56 (5.50)	11.76 (3.67)
Body fat (%), female; mean (SD)	25.50 (6.33)	24.47 (3.27)
Age of first cannabis use; mean (SD)	17.67 (2.46)	16.71 (2.02)
Years of cannabis use; median (IQR)	5.50 (6.5)	5.00 (3.00)
Cannabis use occasions in last year; median (IQR)	5.00 (6.00)	3.00 (7.00)

Abbreviations: BMI, body mass index; IQR, interquartile range.

### Attentional bias task

**FIGURE 2:**
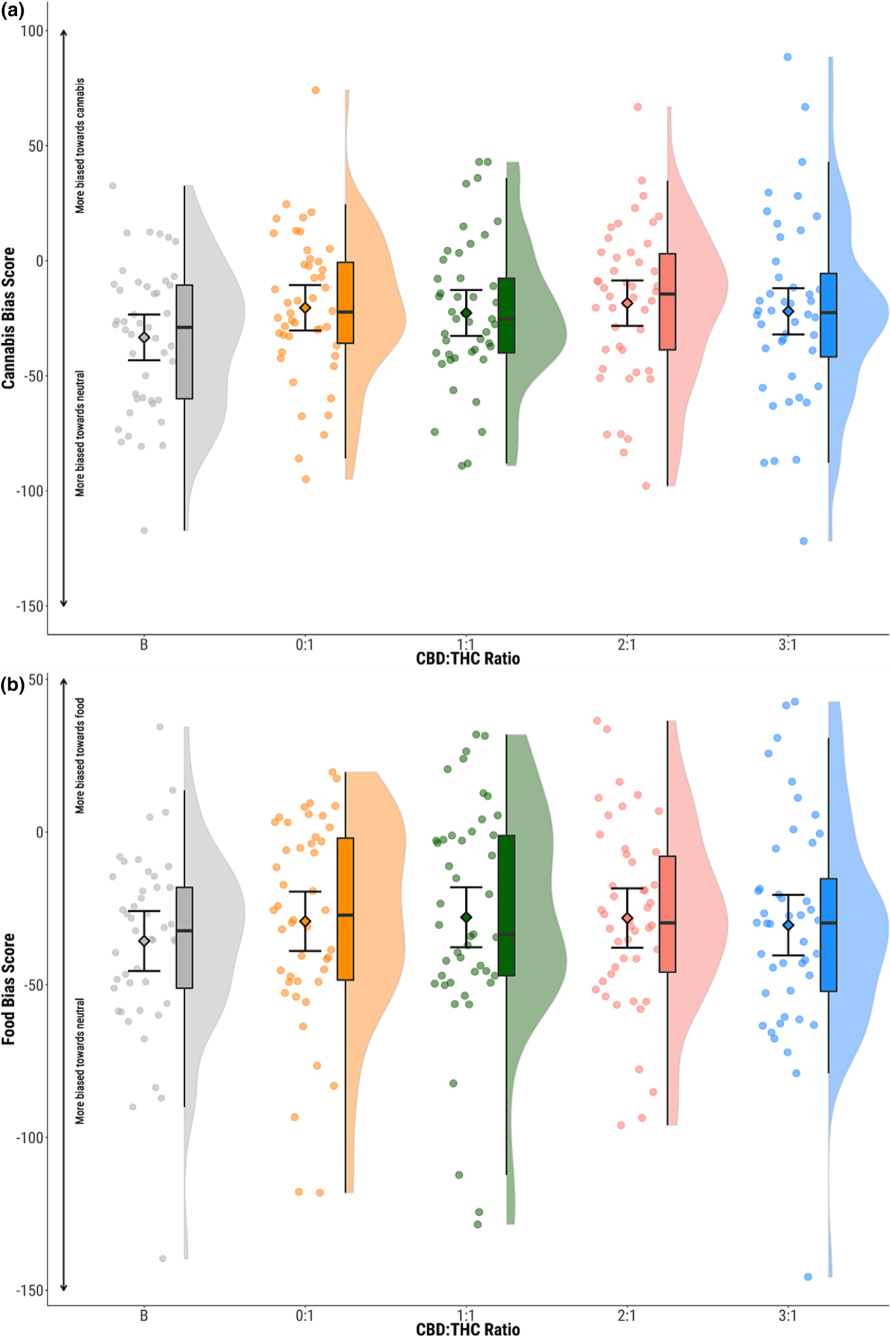
Attentional bias to (a) cannabis and (b) food stimuli. Circles show individual data points, diamonds show mean values, boxplots show median and interquartile range and half violin plots show distribution of participant scores. Baseline (B; grey), CBD:THC ratios 0:1 (orange) 1:1 (green); 2:1 (pink); 3:1 (blue). CBD, cannabidiol; THC, delta‐9‐tetrahydrocannabinol.

Overall, mean bias score values were negative, indicating that participants tended to be more biased toward neutral stimuli than cannabis (mean = −23.37, SD = 34.06) or food stimuli (mean = −30.32, SD = 33.27). *n* = 3 data points were removed (*n* = 2 biased toward neutral; *n* = 1 biased toward cannabis) as outliers and imputed in the cannabis condition and *n* = 0 in the food condition. Participants were significantly more biased toward cannabis stimuli in the 0:1 condition compared to baseline (Figure 2, Table 2). This effect was not maintained when restricting to short or long durations of stimuli presentation. There was no significant difference in bias for food stimuli between baseline and the THC‐only condition (Figure 2, Table 2). Similarly, no effect was seen when restricting to short or long durations of stimuli presentation (Figure S1).

**TABLE 2:** Test results for 0:1‐baseline condition on attentional bias and picture rating task performance.

Outcome	Mean difference (95% CIs)	Cohen's d	Paired *t* test
Attentional bias to cannabis (all trials)	12.2 (1.20, 23.3)	0.41	*t*_44_ = 2.23, *P* = 0.03
Attentional bias to cannabis (short duration)	15.1 (−3.2, 33.4)	0.38	*t*_44_ = 1.66, *P* = 0.10
Attentional bias to cannabis (long duration)	9.8 (−7.0, 26.6)	0.26	*t*_44_ = −1.18, *P* = 0.25
Attentional bias to food (all trials)	6.3 (−5.4, 18.0)	0.21	*t*_44_ = −1.08, *P* = 0.29
Attentional bias to food (short duration)	11.3 (−3.0, 25.7)	0.27	*t*_44_ = −1.59, *P* = 0.12
Attentional bias to food (long duration)	0.3 (−11.7, 12.3)	0.02	*t*_44_ = −0.05, *P* = 0.96
Picture rating cannabis	0.3 (−0.1, 0.7)	0.23	*t*_44_ = −1.59, *P* = 0.12
Picture rating food	0.3 (−0.02, 0.7)	0.21	*t*_44_ = −1.89, *P* = 0.06

No effects of CBD:THC ratio were seen on bias scores for cannabis or food across any duration or congruency of stimulus presentation (*P* > 0.05) (Figure 2, Table 3, Figures S1 and S2 and Tables S2 and S3). This null effect was maintained when including previously excluded trial sessions because of reaction time or incorrect responses (*P* > 0.05) (Figure S3 and Table S4).

**TABLE 3:** Results of linear mixed models of CBD:THC ratio on attentional bias and picture rating task performance.

Contrast	Estimated marginal mean difference	Lower 95% CI	Upper 95% CI	*P* value
	Attentional bias cannabis			
0:1–1:1	1.657	−10.777	14.092	0.993
0:1–2:1	−1.991	−14.344	10.363	0.988
0:1–3:1	2.327	−10.192	14.846	0.982
1:1–2:1	−3.648	−16.083	8.787	0.935
1:1–3:1	0.670	−11.931	13.271	1.000
2:1–3:1	4.318	−8.202	16.837	0.899
	Attentional bias food			
0:1–1:1	−1.203	−14.547	12.141	0.998
0:1–2:1	−1.079	−14.340	12.181	0.998
0:1–3:1	1.247	−12.183	14.677	0.998
1:1–2:1	0.123	−13.221	13.467	1.000
1:1–3:1	2.450	−11.063	15.963	0.983
2:1–3:1	2.327	−11.104	15.757	0.985
	Picture rating cannabis			
0:1–1:1	0.330	0.041	0.620	0.104
0:1–2:1	0.197	−0.093	0.486	0.521
0:1–3:1	0.137	−0.153	0.426	0.778
1:1–2:1	−0.133	−0.423	0.156	0.790
1:1–3:1	−0.194	−0.483	0.096	0.535
2:1–3:1	−0.060	−0.350	0.229	0.975
	Picture rating food			
0:1–1:1	0.214	−0.134	0.562	0.604
0:1–2:1	0.126	−0.221	0.474	0.884
0:1–3:1	−0.032	−0.380	0.315	0.998
1:1–2:1	−0.087	−0.435	0.260	0.958
1:1–3:1	−0.246	−0.594	0.102	0.486
2:1–3:1	−0.159	−0.506	0.189	0.795

No cumulative THC effect was seen on bias scores for cannabis or food across any duration of stimuli presentation (*P* > 0.05) (Figure S4 and Table S5). This was unchanged after adding number of days since the previous experimental visit into the model (*P* > 0.05).

When analyses were re‐run without imputed values, effects were all consistent with these results.

### Picture rating task

**FIGURE 3:**
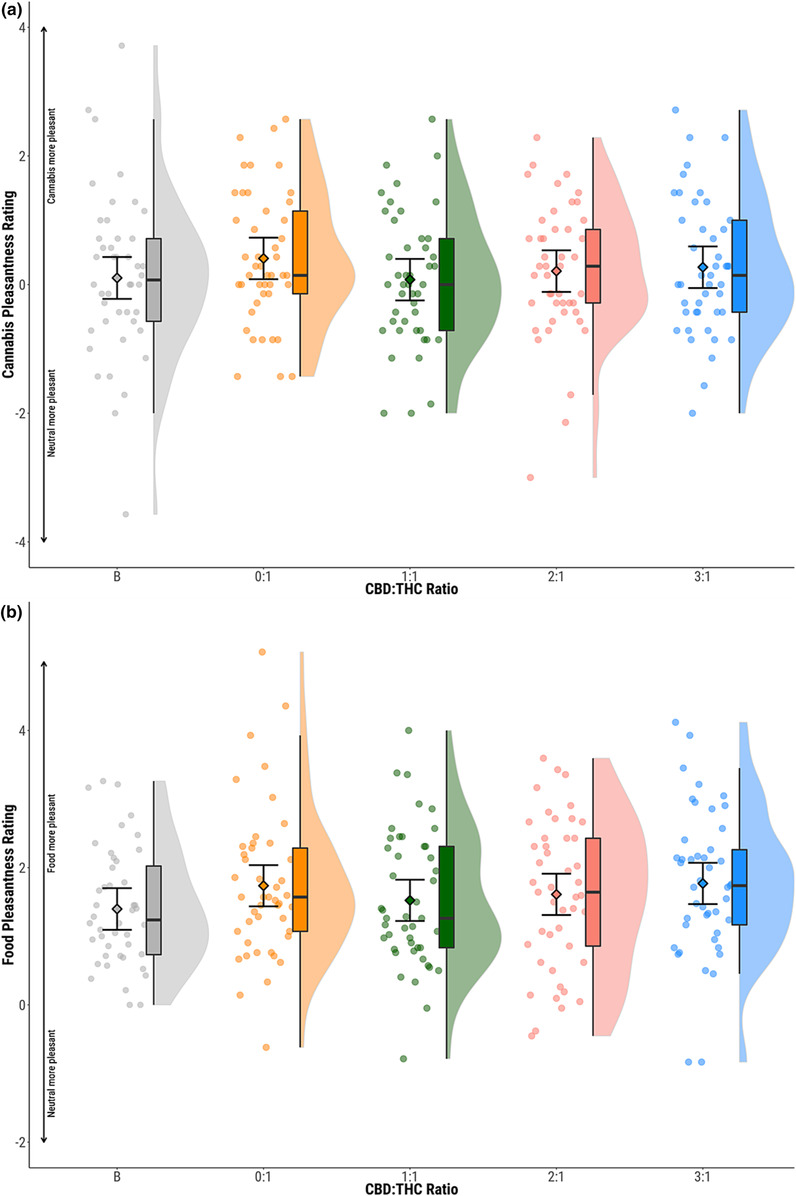
Pleasantness ratings of (a) cannabis and (b) food stimuli. Circles show individual data points, diamonds show mean values, boxplots show median and interquartile range and half violin plots show distribution of participant scores. Baseline (B; grey), CBD:THC ratios 0:1 (orange) 1:1 (green); 2:1 (pink); 3:1 (blue). CBD, cannabidiol; THC, delta‐9‐tetrahydrocannabinol.

Mean picture rating scores indicated greater preference for cannabis (mean = 0.22, SD = 1.09) and food stimuli (mean = 1.61, SD = 1.02) compared to neutral. There were no significant differences between baseline and 0:1 conditions for cannabis or food stimuli (Figure 3, Table 2).

No effects of CBD:THC ratio was seen on picture ratings for cannabis or food stimuli (*P* > 0.05) (Figure 3, Table 3).

No cumulative THC effect was seen on picture ratings for cannabis or food stimuli (*P* > 0.05) (Figure S5 and Table S6). This was unchanged after adding number of days since the previous experimental visit into the model (*P* > 0.05).

### Correlations with plasma THC and CBD concentrations

There were no differences in THC levels across drug conditions, and a significant dose response relationship for CBD levels across drug conditions—validating the selected CBD:THC ratios. For comprehensive pharmacokinetics see Englund *et al*. [19] There were no significant correlations between attentional bias toward cannabis stimuli and either peak plasma THC (*r* = −0.06, *P* = 0.43) (Figure S6), THC AUC (*r* = −0.08, *P* = 0.31) (Figure S6), peak plasma CBD (*r* = −0.01, *P* = 0.88) (Figure S6) or CBD AUC (*r* = −0.02, *P* = 0.79) (Figure S6). Similarly, there were no significant correlations between the above plasma measures on attentional bias toward food stimuli or picture ratings for cannabis or food stimuli (*P* > 0.05) (Figures S6 and S7).

When analyses were re‐run without imputed values, effects were all consistent with these results.

## DISCUSSION

This is the first and best‐controlled study, to our knowledge, to explore the effects of cannabis with varying CBD:THC ratios on cognitive mechanisms underpinning CUD in healthy infrequent cannabis users. We found that acute cannabis inhalation induced significant attentional bias toward cannabis compared to baseline in users with no previous history of substance use disorder. CBD did not attenuate this effect at any ratio tested. However, cannabis intoxication did not increase ratings of explicit liking of cannabis.

Our results may suggest that acute intoxication with THC is biasing participants toward cannabis without them being consciously aware of it, potentially increasing the susceptibility toward CUD. This is unlikely to be merely an effect of slowed reaction time because of the intoxicating effects of cannabis because bias scores are relative to reaction time for neutral images. Moreover, the effect was specific for cannabis, but not food stimuli and there was a lack of order effects. It is notable this effect was not present when restricting to short duration trials only, which should represent less conscious control and therefore, index purer implicit wanting of cannabis.

Because our study administered several doses of THC to participants across four visits, we were also able to compare whether there was an effect of THC plasma concentration or cumulative THC dose on attentional bias. We found no such effect. Although previous studies have found robust links between cannabis potency, frequency of cannabis use and CUD [3, 5], our lack of an effect of cumulative THC exposure may indicate that using infrequently (less than weekly) may mitigate against changes in cognitive processes associated with CUD, regardless of cannabis potency. This mirrors evidence for cannabis use as a risk factor for psychosis, which suggests increases in risk only becomes evidence at higher levels of frequency [29, 30].

Contrary to our hypothesis, the addition of CBD, at any ratio, had no impact on either attentional bias or explicit liking of cannabis or food stimuli. These findings contrast with those of Morgan *et al*. [17] where regular cannabis users showed reduced attentional bias toward cannabis if they regularly used cannabis with high CBD:THC ratios compared to those who used low CBD:THC ratio cannabis. This attenuation was seen both as a trait effect when sober (on a drug‐free visit) and acutely when intoxicated with their own cannabis (on an intoxication visit). On average, the high CBD:THC cannabis was a 1:3 CBD:THC ratio, much lower than the lowest CBD‐containing CBD:THC ratio in our study. This could suggest that the protective effects of CBD are only seen through longer‐term, regular use. Alternatively, this may be because this was a naturalistic study design where THC dose was not fixed, with participants instead using an amount of their cannabis they deemed sufficient for them to typically become intoxicated, compared to our controlled experimental design. It also needs to be borne in mind that, because THC and CBD are both the product of cannabigerol (or rather their corresponding carboxylic acids) within the cannabis plant [31], naturally higher CBD‐containing cannabis will have lower concentrations of THC. This could suggest that the protective effects of the high CBD:THC ratio cannabis may have been because of regular use of lower potency cannabis (7.7% compared to 11.9%), rather than the higher CBD content (2.6% compared to 0.1%). As people typically do not effectively titrate their use according to potency [7], this discrepancy would likely translate to lower THC doses consistently being used.

Other studies, such as Hindocha *et al*. [32] and Hurd *et al*. [33, 34], have also showed CBD having an ameliorating effect. However, these studies were conducted on participants with either nicotine or opioid use disorder, and CBD was administered as an oral pre‐treatment at much higher doses than in the present study.

Our study benefits from many strengths such as being relatively high‐powered because of the cross‐over double‐blind placebo‐controlled design, with *n* = 230 study visits, and the exploration of a potential dose‐response relationship of CBD. We explored the most available CBD:THC ratios of cannabis sold both in legal and illegal markets [35], and administered these in a randomised, double‐blind, counter‐balanced order. However, our findings may not generalise to CBD:THC ratios not tested in this study. A further strength of our study was the collection of blood samples for pharmacokinetic analysis that allowed us to investigate plasma concentration‐response to account for bioavailability variation. Limitations of our study include the lack of a placebo condition for the THC element itself, which was not included as the main aim of the study was to explore the putative protective effects of CBD rather than the effects of THC. Non‐inclusion of a placebo condition for THC also avoided over‐burdening the participants because they were already required to attend five whole‐day study visits. Because of fewer trials for short and long duration of stimulus presentation compared to overall, there is less precision in estimates. Greater power may be needed to understand potential differences. We additionally used a per‐protocol analysis as outcome data was limited for individuals who dropped out of the study, which can lead to selection biases. However, this is less of an issue as we are interested in within‐subject effects with each subject acting as their own control. We did not have a perfectly counterbalanced order of CBD:THC ratios, which could introduce bias. However, there was no effect of visit order, as seen in our cumulative THC analyses so any bias is likely negligible. Additionally, we were unable to perform intent‐to‐treat analyses because of the high proportion of drop‐outs who did not provide any outcome data. Future studies should explore differences in attentional bias comparing infrequent and frequent cannabis users, as well as exploring the effects of different doses of THC using both inhaled and oral routes of administration.

In summary, we found that, compared to sober baseline conditions, the inhalation of THC induced attentional bias toward cannabis images in the absence of conscious liking of such images in healthy participants who use cannabis infrequently. This suggests that even infrequent users may show cognitive processes associated with liability toward CUD. We also found that, at the concentrations normally found in legal and illegal cannabis, the presence of CBD in cannabis is unlikely to influence this.

## AUTHOR CONTRIBUTIONS

**Dominic Oliver:** Conceptualization (equal); data curation (equal); formal analysis (lead); investigation (lead); methodology (equal); project administration (lead); supervision (equal); validation (lead); visualization (lead); writing—original draft (lead); writing—review and editing (lead). **Amir Englund:** Conceptualization (lead); data curation (lead); formal analysis (lead); funding acquisition (lead); investigation (lead); methodology (lead); project administration (lead); resources (lead); supervision (lead); validation (lead); visualization (lead); writing—original draft (equal); writing—review and editing (lead). **Edward Chesney:** Data curation (equal); formal analysis (equal); investigation (equal); methodology (equal); project administration (equal); supervision (equal); validation (equal); visualization (equal); writing—original draft (supporting); writing—review and editing (supporting). **Lucy Chester:** Data curation (equal); formal analysis (equal); investigation (equal); methodology (equal); project administration (equal); supervision (equal); validation (equal); visualization (equal); writing—original draft (supporting); writing—review and editing (supporting). **Jack Wilson:** Data curation (equal); investigation (equal); methodology (equal); project administration (equal); supervision (equal); validation (equal); visualization (equal); writing—original draft (supporting); writing—review and editing (supporting). **Simina Sovi:** Data curation (equal); formal analysis (equal); investigation (supporting); project administration (supporting); validation (supporting); visualization (supporting); writing—original draft (supporting); writing—review and editing (supporting). **Stina Wigroth:** Data curation (equal); formal analysis (supporting); validation (supporting); visualization (supporting); writing—original draft (supporting); writing—review and editing (supporting). **John Hodsoll:** Conceptualization (equal); data curation (equal); formal analysis (equal); funding acquisition (supporting); methodology (equal); software (equal); validation (equal); visualization (equal); writing—original draft (supporting); writing—review and editing (supporting). **John Strang:** Investigation (equal); methodology (supporting); project administration (supporting); supervision (equal); validation (supporting); visualization (supporting); writing—original draft (supporting); writing—review and editing (supporting). **Robin M. Murray:** Conceptualization (lead); funding acquisition (lead); investigation (equal); methodology (lead); resources (equal); supervision (equal); writing—original draft (supporting); writing—review and editing (supporting). **Tom P. Freeman:** Conceptualization (lead); data curation (supporting); formal analysis (supporting); funding acquisition (lead); investigation (supporting); methodology (lead); project administration (supporting); resources (equal); supervision (supporting); validation (supporting); visualization (supporting); writing—original draft (supporting); writing—review and editing (supporting). **Paolo Fusar‐Poli:** Formal analysis (equal); investigation (equal); methodology (supporting); project administration (supporting); supervision (lead); writing—original draft (equal); writing—review and editing (equal). **Philip McGuire:** Conceptualization (lead); data curation (equal); formal analysis (equal); funding acquisition (lead); investigation (equal); methodology (lead); project administration (equal); resources (lead); supervision (lead); validation (equal); visualization (equal); writing—original draft (equal); writing—review and editing (equal).

## DECLARATION OF INTERESTS

A.E. has received speakers' honoraria from GW, Otsuka and Lundbeck Pharmaceuticals. R.M.M. has received speakers' honoraria from Lundbeck, Sunovian, Otsuka and Janssen. J.S. has undertaken research supported financially by various pharmaceutical companies, but this has not involved studies of cannabis or cannabis‐related products. All remaining authors report no conflicting interests.

## CLINICAL TRIAL REGISTRATION

The study was registered on Open Science Framework (https://osf.io/kt3f7) and clinicaltrials.gov (NCT05170217).

## Supporting information


**Table S1.** Weight of Bedrocan, Bedrolite and placebo cannabis in each CBD:THC ratio.
**Table S2**. Results of linear mixed models for attentional bias task.
**Figure S1**. Attentional bias stratified by durations of stimulus presentation.
**Table S3**. Results of linear mixed models for durations of stimulus presentation on attentional bias.
**Figure S2**. Attentional bias stratified by congruency of stimulus presentation.
**Table S4**. Results of linear mixed models for congruency of stimulus presentation on attentional bias.
**Figure S3**. Sensitivity analysis: attentional bias with re‐included excluded trials due to reaction time and incorrect responses.
**Table S5.** Sensitivity analysis: attentional bias with re‐included excluded trials due to reaction time and incorrect responses.
**Figure S4.** Cumulative THC effect on attentional bias.
**Table S6.** Results of linear mixed models for cumulative THC effect on attentional bias.
**Table S7.** Results of linear mixed models for picture rating task.
**Figure S5.** Cumulative THC effect on picture rating.
**Table S8.** Results of linear mixed models for cumulative THC effect on picture rating.
**Figure S6.** Correlations between plasma cannabinoids and attentional bias.
**Figure S7.** Correlations between plasma cannabinoids and picture rating.
**Figure S8.** Study CONSORT flow diagram.

## Data Availability

The data that support the findings of this study are available on request from the corresponding author. The data are not publicly available due to privacy or ethical restrictions.
