# Supplementary material for: Cannabidiol does not attenuate acute delta‐9‐tetrahydrocannabinol‐induced attentional bias in healthy volunteers: A randomised, double‐blind, cross‐over study
Source: Addiction. 2023 Oct 11;119(2):322–33. doi: 10.1111/add.16353 (PMC10952555; doi:10.1111/add.16353)

**Supplementary Material**

Oliver D, Englund A, Chesney E, Chester L, Wilson J, Sovi S, Wigroth S, Hodsoll J, Strang J, Murray R, Freeman T, Fusar-Poli P, McGuire P. Cannabidiol does not attenuate acute delta-9-tetrahydrocannabinol-induced attentional bias changes in healthy volunteers: A randomised, double-blind, crossover study

**eTable 1** Weight of Bedrocan, Bedrolite and placebo cannabis in each CBD:THC ratio

**eTable 2** Results of linear mixed models for attentional bias task

**eFigure 1** Attentional bias stratified by durations of stimulus presentation

**eTable 3** Results of linear mixed models for durations of stimulus presentation on attentional bias

**eFigure 2** Attentional bias stratified by congruency of stimulus presentation

**eTable 4** Results of linear mixed models for congruency of stimulus presentation on attentional bias

**eFigure 3** Sensitivity analysis: attentional bias with re-included excluded trials due to reaction time and incorrect responses

**eTable 5** Sensitivity analysis: attentional bias with re-included excluded trials due to reaction time and incorrect responses

**eFigure 4** Cumulative THC effect on attentional bias

**eTable 6** Results of linear mixed models for cumulative THC effect on attentional bias

**eTable 7** Results of linear mixed models for picture rating task

**eFigure 5** Cumulative THC effect on picture rating

**eTable 8** Results of linear mixed models for cumulative THC effect on picture rating

**eFigure 6** Correlations between plasma cannabinoids and attentional bias

**eFigure 7** Correlations between plasma cannabinoids and picture rating

**eFigure 8** Study CONSORT flow diagram

**eTable 1** Weight of Bedrocan, Bedrolite and placebo cannabis in each CBD:THC ratio

| CBD:THC ratio | 0:1 | 1:1 | 2:1 | 3:1 |
| --- | --- | --- | --- | --- |
| THC dose (mg) | 10 | 10 | 10 | 10 |
| CBD dose (mg) | 0 | 10 | 20 | 30 |
| Bedrocan cannabis (mg) | 44.2 | 42.5 | 40.7 | 38.9 |
| Bedrolite cannabis (mg) | 0.0 | 132.8 | 266.1 | 399.5 |
| Placebo cannabis (mg) | 394.2 | 263.1 | 131.6 | 0.0 |

**eTable 2** Results of linear mixed models for attentional bias task

| **Contrast** | **Estimated marginal mean difference** | **Lower 95% CI** | **Upper 95% CI** | **p-value** |
| --- | --- | --- | --- | --- |
|  | **Cannabis** |  |  |  |
| 0:1 - 1:1 | 1.657 | -10.777 | 14.092 | 0.993 |
| 0:1 - 2:1 | -1.991 | -14.344 | 10.363 | 0.988 |
| 0:1 - 3:1 | 2.327 | -10.192 | 14.846 | 0.982 |
| 1:1 - 2:1 | -3.648 | -16.083 | 8.787 | 0.935 |
| 1:1 - 3:1 | 0.670 | -11.931 | 13.271 | 1.000 |
| 2:1 - 3:1 | 4.318 | -8.202 | 16.837 | 0.899 |
|  | **Food** | | | |
| 0:1 - 1:1 | -1.203 | -14.547 | 12.141 | 0.998 |
| 0:1 - 2:1 | -1.079 | -14.340 | 12.181 | 0.998 |
| 0:1 - 3:1 | 1.247 | -12.183 | 14.677 | 0.998 |
| 1:1 - 2:1 | 0.123 | -13.221 | 13.467 | 1.000 |
| 1:1 - 3:1 | 2.450 | -11.063 | 15.963 | 0.983 |
| 2:1 - 3:1 | 2.327 | -11.104 | 15.757 | 0.985 |

**eFigure 1** Attentional bias stratified by durations of stimulus presentation


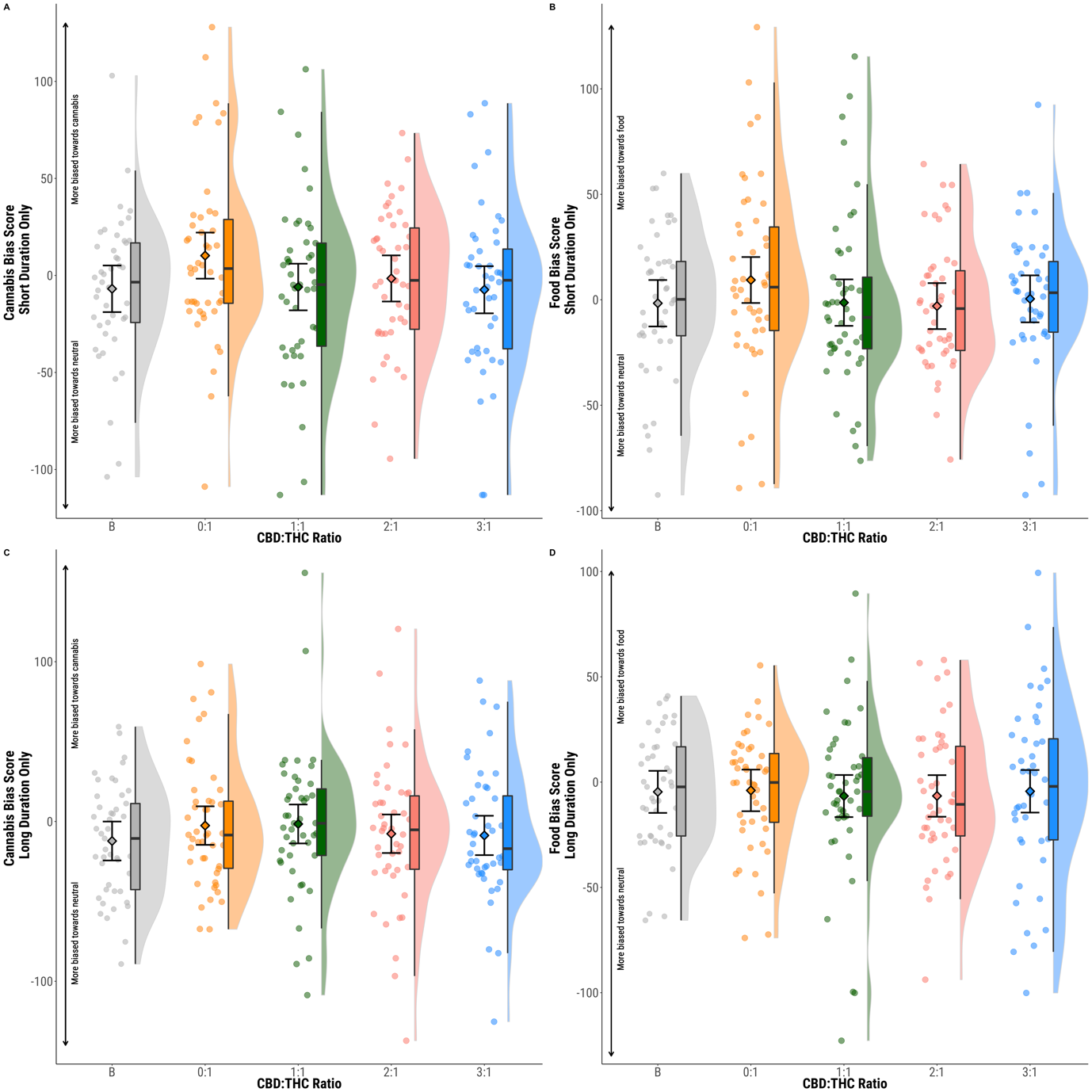


| **eTable 3** Results of linear mixed models for durations of stimulus presentation on attentional bias | | | | |
| --- | --- | --- | --- | --- |
| **Contrast** | **Estimated marginal mean difference** | **Lower 95% CI** | **Upper 95% CI** | **p-value** |
|  | **Cannabis Short** |  |  |  |
| 0:1 - 1:1 | 14.541 | -1.804 | 30.886 | 0.282 |
| 0:1 - 2:1 | 10.170 | -6.077 | 26.417 | 0.590 |
| 0:1 - 3:1 | 11.297 | -5.150 | 27.744 | 0.512 |
| 1:1 - 2:1 | -4.371 | -20.716 | 11.974 | 0.949 |
| 1:1 - 3:1 | -3.244 | -19.788 | 13.301 | 0.979 |
| 2:1 - 3:1 | 1.128 | -15.319 | 17.575 | 0.999 |
|  | **Cannabis Long** | | | |
| 0:1 - 1:1 | 0.213 | -16.220 | 16.645 | 1.000 |
| 0:1 - 2:1 | 5.113 | -11.214 | 21.440 | 0.922 |
| 0:1 - 3:1 | 6.237 | -10.305 | 22.779 | 0.872 |
| 1:1 - 2:1 | 4.900 | -11.532 | 21.333 | 0.932 |
| 1:1 - 3:1 | 6.024 | -10.623 | 22.671 | 0.885 |
| 2:1 - 3:1 | 1.124 | -15.418 | 17.666 | 0.999 |
|  | **Food Short** |  |  |  |
| 0:1 - 1:1 | 8.876 | -6.187 | 23.938 | 0.636 |
| 0:1 - 2:1 | 12.362 | -2.605 | 27.329 | 0.347 |
| 0:1 - 3:1 | 2.043 | -13.120 | 17.205 | 0.993 |
| 1:1 - 2:1 | 3.486 | -11.577 | 18.549 | 0.966 |
| 1:1 - 3:1 | -6.833 | -22.091 | 8.425 | 0.804 |
| 2:1 - 3:1 | -10.319 | -25.481 | 4.843 | 0.520 |
|  | **Food Long** | | | |
| 0:1 - 1:1 | -0.805 | -15.293 | 13.683 | 0.999 |
| 0:1 - 2:1 | 2.623 | -11.780 | 17.026 | 0.983 |
| 0:1 - 3:1 | 0.414 | -14.162 | 14.989 | 1.000 |
| 1:1 - 2:1 | 3.428 | -11.060 | 17.916 | 0.964 |
| 1:1 - 3:1 | 1.219 | -13.441 | 15.878 | 0.998 |
| 2:1 - 3:1 | -2.209 | -16.785 | 12.366 | 0.990 |

**eFigure 2** Attentional bias stratified by congruency of stimulus presentation


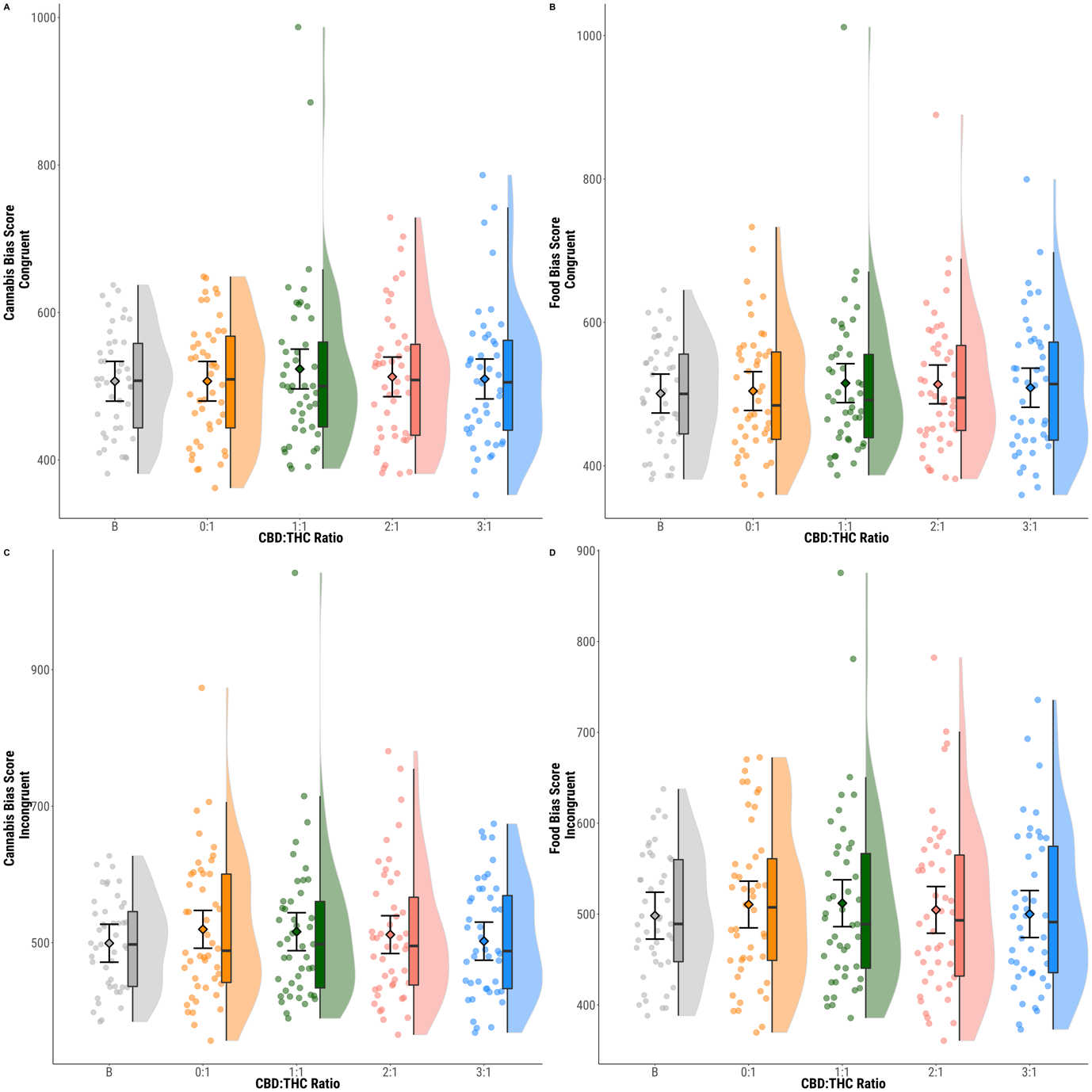


**eTable 4** Results of linear mixed models for congruency of stimulus presentation on attentional bias

| **Contrast** | **Estimated marginal mean difference** | **Lower 95% CI** | **Upper 95% CI** | **p-value** |
| --- | --- | --- | --- | --- |
|  | **Cannabis Congruent** |  |  |  |
| 0:1 - 1:1 | -16.826 | -36.154 | 2.501 | 0.301 |
| 0:1 - 2:1 | -5.909 | -25.097 | 13.280 | 0.925 |
| 0:1 - 3:1 | -2.411 | -21.883 | 17.061 | 0.995 |
| 1:1 - 2:1 | 10.918 | -8.410 | 30.246 | 0.667 |
| 1:1 - 3:1 | 14.415 | -5.197 | 34.028 | 0.452 |
| 2:1 - 3:1 | 3.497 | -15.974 | 22.969 | 0.984 |
|  | **Cannabis Incongruent** | | | |
| 0:1 - 1:1 | 3.499 | -17.921 | 24.920 | 0.988 |
| 0:1 - 2:1 | 7.769 | -13.498 | 29.036 | 0.883 |
| 0:1 - 3:1 | 18.127 | -3.452 | 39.707 | 0.332 |
| 1:1 - 2:1 | 4.269 | -17.151 | 25.690 | 0.978 |
| 1:1 - 3:1 | 14.628 | -7.107 | 36.363 | 0.529 |
| 2:1 - 3:1 | 10.358 | -11.221 | 31.938 | 0.769 |
|  | **Food Congruent** |  |  |  |
| 0:1 - 1:1 | -11.185 | -30.850 | 8.481 | 0.662 |
| 0:1 - 2:1 | -9.290 | -28.814 | 10.235 | 0.773 |
| 0:1 - 3:1 | -4.068 | -23.880 | 15.744 | 0.976 |
| 1:1 - 2:1 | 1.895 | -17.771 | 21.560 | 0.997 |
| 1:1 - 3:1 | 7.117 | -12.838 | 27.072 | 0.890 |
| 2:1 - 3:1 | 5.222 | -14.590 | 25.034 | 0.951 |
|  | **Food Incongruent** | | | |
| 0:1 - 1:1 | -1.296 | -17.522 | 14.930 | 0.999 |
| 0:1 - 2:1 | 5.919 | -10.189 | 22.028 | 0.881 |
| 0:1 - 3:1 | 11.025 | -5.322 | 27.373 | 0.528 |
| 1:1 - 2:1 | 7.215 | -9.011 | 23.441 | 0.807 |
| 1:1 - 3:1 | 12.321 | -4.146 | 28.787 | 0.436 |
| 2:1 - 3:1 | 5.106 | -11.242 | 21.453 | 0.923 |

**eFigure 3** Sensitivity analysis: attentional bias with re-included excluded trials due to reaction time and incorrect responses


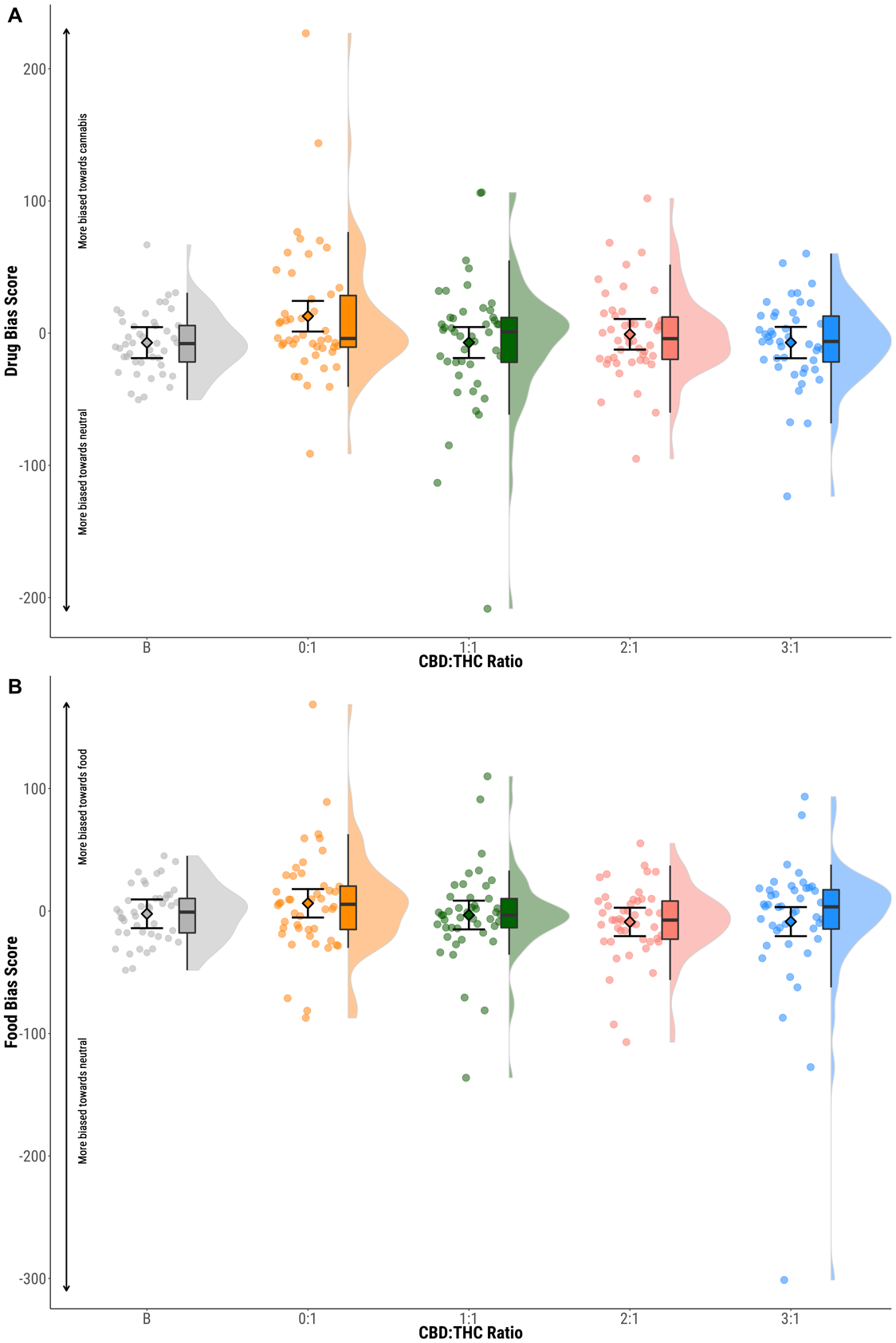


**eTable 5** Sensitivity analysis: attentional bias with re-included excluded trials due to reaction time and incorrect responses

| **Contrast** | **Estimated marginal mean difference** | **Lower 95% CI** | **Upper 95% CI** | **p-value** |
| --- | --- | --- | --- | --- |
|  | **Cannabis** |  |  |  |
| 0:1 - 1:1 | 19.937 | 1.931 | 37.943 | 0.120 |
| 0:1 - 2:1 | 13.677 | -4.225 | 31.580 | 0.417 |
| 0:1 - 3:1 | 19.972 | 1.859 | 38.085 | 0.123 |
| 1:1 - 2:1 | -6.260 | -24.266 | 11.746 | 0.897 |
| 1:1 - 3:1 | 0.035 | -18.181 | 18.250 | 1.000 |
| 2:1 - 3:1 | 6.294 | -11.819 | 24.407 | 0.897 |
|  | **Food** | | | |
| 0:1 - 1:1 | 9.641 | -7.874 | 27.155 | 0.685 |
| 0:1 - 2:1 | 15.209 | -2.201 | 32.619 | 0.298 |
| 0:1 - 3:1 | 14.941 | -2.683 | 32.565 | 0.324 |
| 1:1 - 2:1 | 5.569 | -11.946 | 23.084 | 0.919 |
| 1:1 - 3:1 | 5.301 | -12.428 | 23.029 | 0.931 |
| 2:1 - 3:1 | -0.268 | -17.892 | 17.356 | 1.000 |

**eFigure 4** Cumulative THC effect on attentional bias


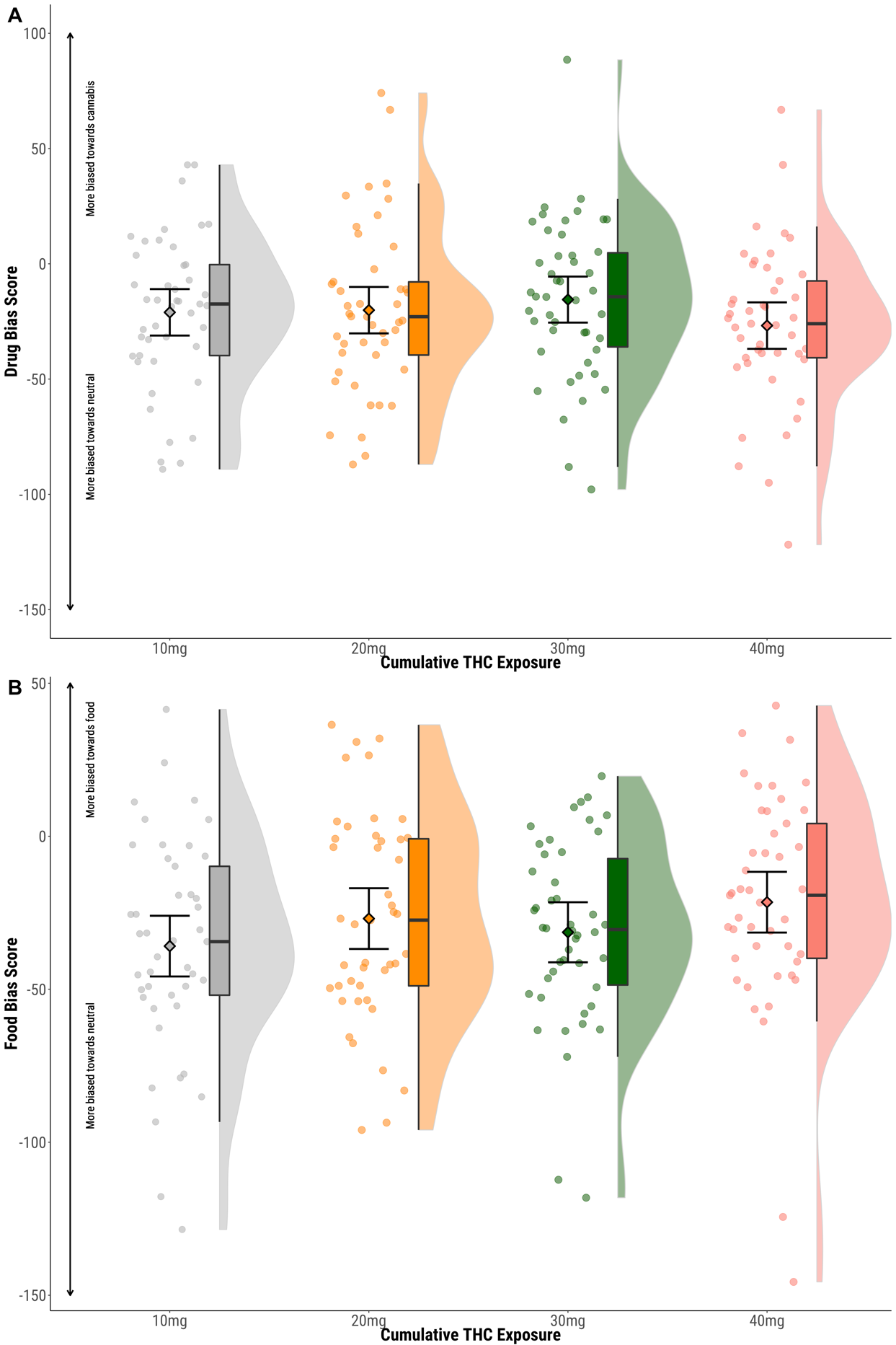


**eTable 6** Results of linear mixed models for cumulative THC effect on attentional bias

| **Contrast** | **Estimated marginal mean difference** | **Lower 95% CI** | **Upper 95% CI** | **p-value** |
| --- | --- | --- | --- | --- |
|  | **Cannabis** |  |  |  |
| 10mg-20mg | -0.406 | -12.808 | 11.995 | 1.000 |
| 10mg – 30mg | -5.680 | -18.000 | 6.641 | 0.790 |
| 10mg – 40mg | 5.067 | -7.334 | 17.468 | 0.844 |
| 20mg – 30mg | -5.273 | -17.594 | 7.047 | 0.824 |
| 20mg – 40mg | 5.473 | -6.928 | 17.874 | 0.811 |
| 30mg – 40mg | 10.746 | -1.574 | 23.067 | 0.299 |
|  | **Food** | | | |
| 10mg-20mg | -8.994 | -22.178 | 4.190 | 0.518 |
| 10mg – 30mg | -4.521 | -17.624 | 8.581 | 0.899 |
| 10mg – 40mg | -14.354 | -27.538 | -1.170 | 0.131 |
| 20mg – 30mg | 4.472 | -8.630 | 17.575 | 0.902 |
| 20mg – 40mg | -5.361 | -18.545 | 7.824 | 0.845 |
| 30mg – 40mg | -9.833 | -22.935 | 3.269 | 0.434 |

**eTable 7** Results of linear mixed models for picture rating task

| **Contrast** | **Estimated marginal mean difference** | **Lower 95% CI** | **Upper 95% CI** | **p-value** |
| --- | --- | --- | --- | --- |
|  | **Cannabis** |  |  |  |
| 0:1 - 1:1 | 0.330 | 0.041 | 0.620 | 0.104 |
| 0:1 - 2:1 | 0.197 | -0.093 | 0.486 | 0.521 |
| 0:1 - 3:1 | 0.137 | -0.153 | 0.426 | 0.778 |
| 1:1 - 2:1 | -0.133 | -0.423 | 0.156 | 0.790 |
| 1:1 - 3:1 | -0.194 | -0.483 | 0.096 | 0.535 |
| 2:1 - 3:1 | -0.060 | -0.350 | 0.229 | 0.975 |
|  | **Food** | | | |
| 0:1 - 1:1 | 0.214 | -0.134 | 0.562 | 0.604 |
| 0:1 - 2:1 | 0.126 | -0.221 | 0.474 | 0.884 |
| 0:1 - 3:1 | -0.032 | -0.380 | 0.315 | 0.998 |
| 1:1 - 2:1 | -0.087 | -0.435 | 0.260 | 0.958 |
| 1:1 - 3:1 | -0.246 | -0.594 | 0.102 | 0.486 |
| 2:1 - 3:1 | -0.159 | -0.506 | 0.189 | 0.795 |

**eFigure 5** Cumulative THC effect on picture rating


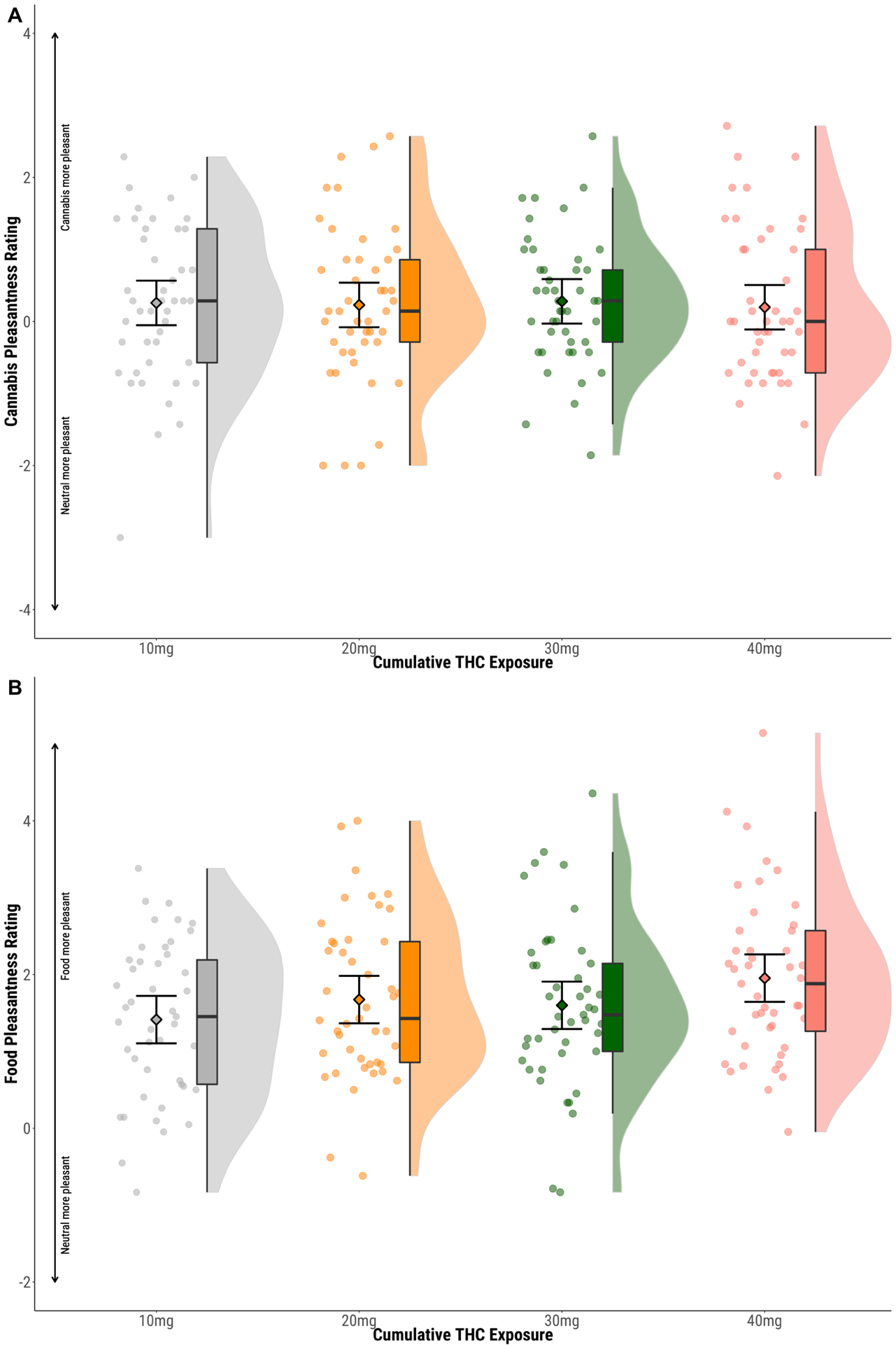


**eTable 8** Results of linear mixed models for cumulative THC effect on picture rating

| **Contrast** | **Estimated marginal mean difference** | **Lower 95% CI** | **Upper 95% CI** | **p-value** |
| --- | --- | --- | --- | --- |
|  | **Cannabis** |  |  |  |
| 10mg-20mg | 0.029 | -0.266 | 0.324 | 0.997 |
| 10mg – 30mg | -0.022 | -0.317 | 0.273 | 0.999 |
| 10mg – 40mg | 0.060 | -0.235 | 0.355 | 0.976 |
| 20mg – 30mg | -0.051 | -0.346 | 0.244 | 0.986 |
| 20mg – 40mg | 0.032 | -0.263 | 0.327 | 0.996 |
| 30mg – 40mg | 0.083 | -0.212 | 0.378 | 0.943 |
|  | **Food** | | | |
| 10mg-20mg | -0.260 | -0.598 | 0.077 | 0.410 |
| 10mg – 30mg | -0.186 | -0.523 | 0.152 | 0.686 |
| 10mg – 40mg | -0.539 | -0.877 | -0.201 | 0.009 |
| 20mg – 30mg | 0.075 | -0.263 | 0.412 | 0.971 |
| 20mg – 40mg | -0.279 | -0.617 | 0.059 | 0.348 |
| 30mg – 40mg | -0.353 | -0.691 | -0.016 | 0.156 |

**eFigure 6** Correlations between plasma cannabinoids and attentional bias


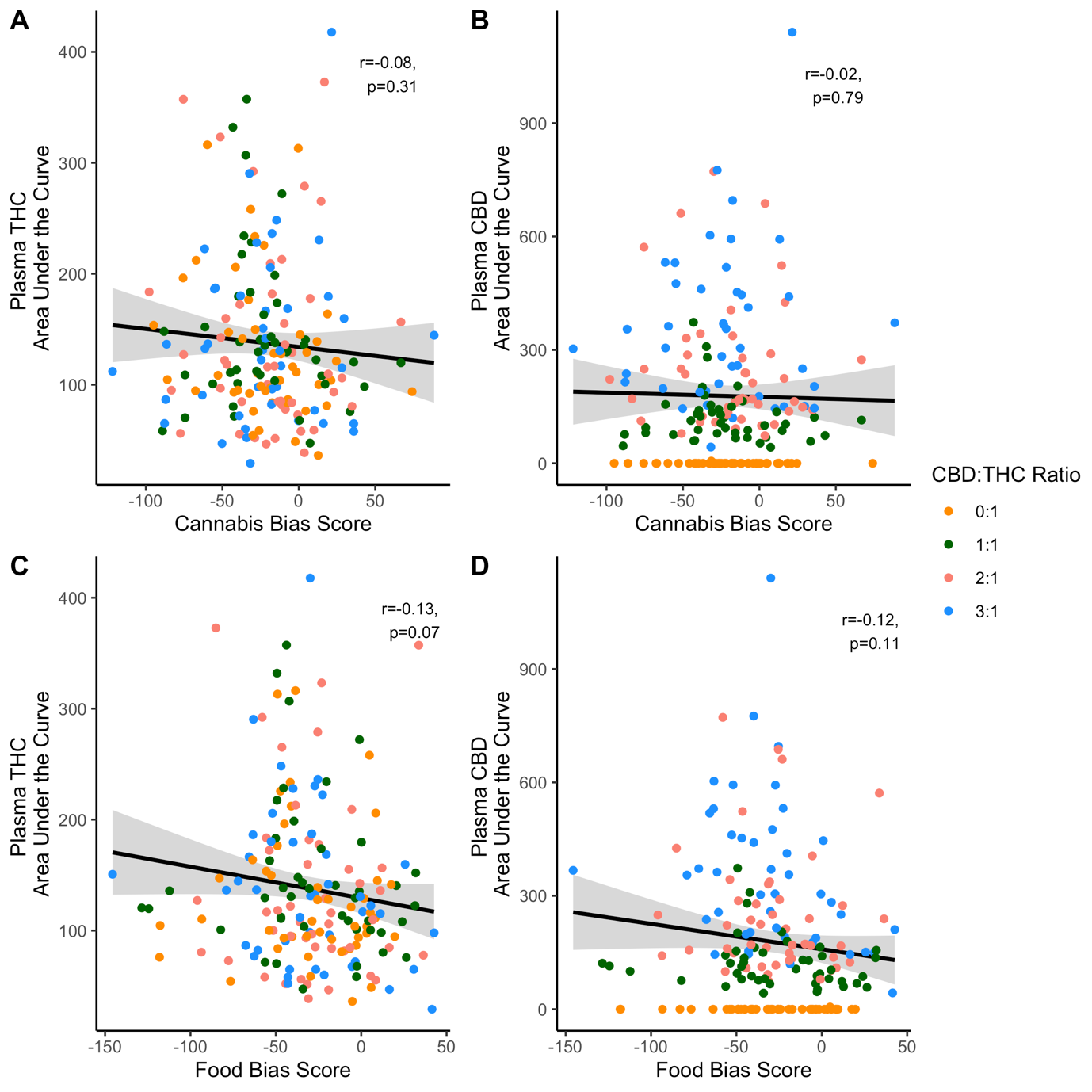


**eFigure 7** Correlations between plasma cannabinoids and picture rating


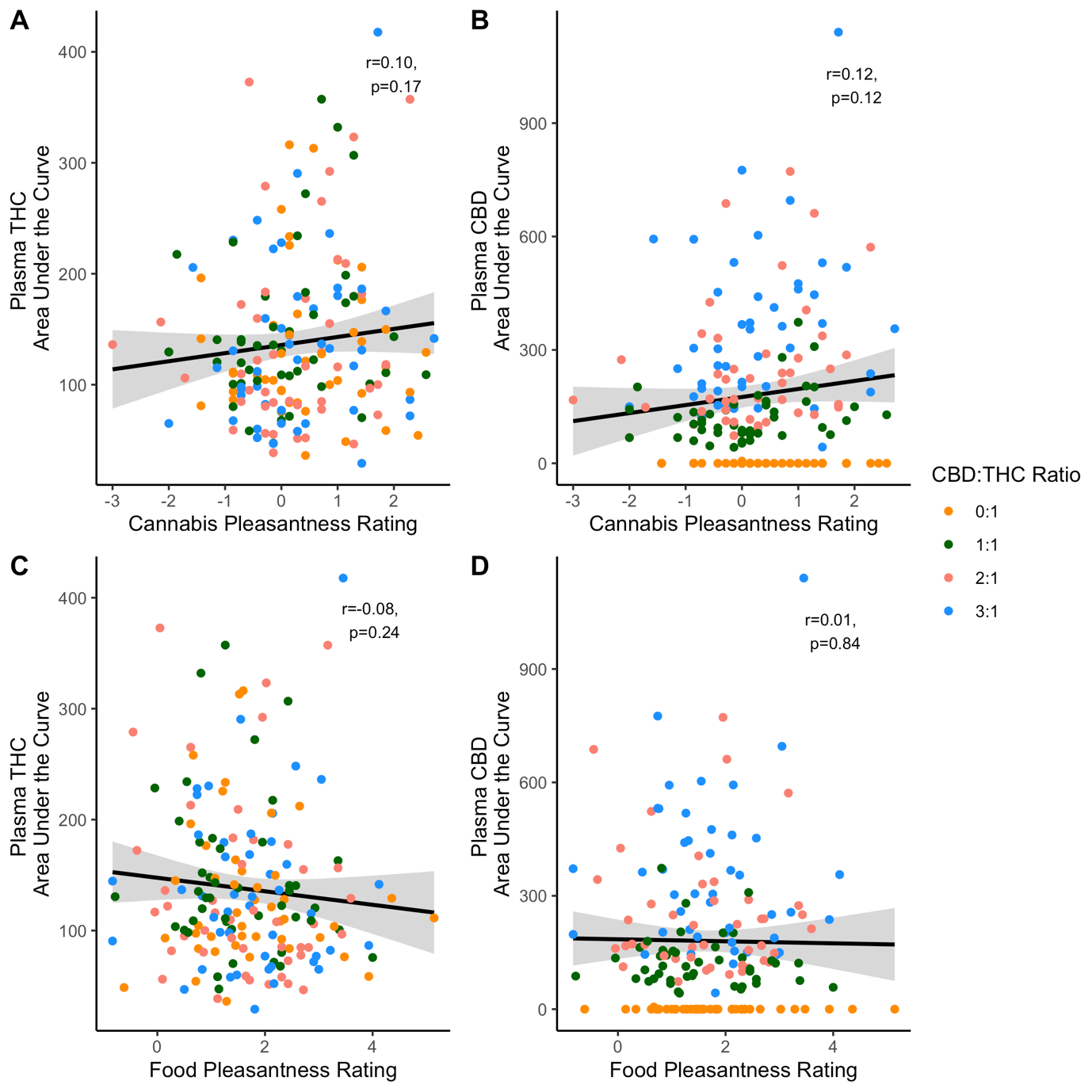


**eFigure 8** Study CONSORT flow diagram


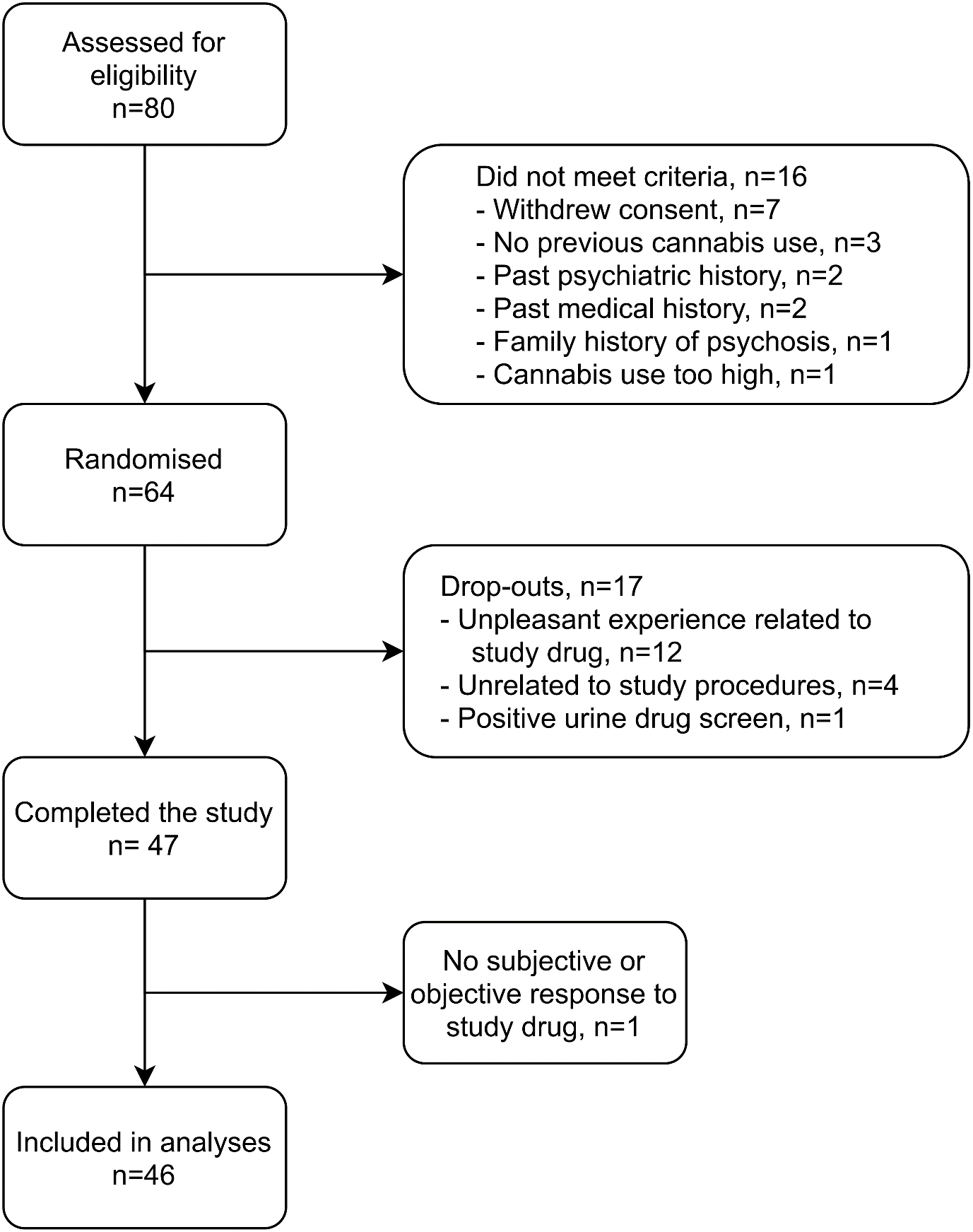

Supplement: Supplementary file 1 — Table S1. Weight of Bedrocan, Bedrolite and placebo cannabis in each CBD:THC ratio. Table S2. Results of linear mixed models for attentional bias task. Figure S1. Attentional bias stratified by durations of stimulus presentation. Table S3. Results of linear mixed models for durations of stimulus presentation on attentional bias. Figure S2. Attentional bias stratified by congruency of stimulus presentation. Table S4. Results of linear mixed models for congruency of stimulus presentation on attentional bias. Figure S3. Sensitivity analysis: attentional bias with re‐included excluded trials due to reaction time and incorrect responses. Table S5. Sensitivity analysis: attentional bias with re‐included excluded trials due to reaction time and incorrect responses. Figure S4. Cumulative THC effect on attentional bias. Table S6. Results of linear mixed models for cumulative THC effect on attentional bias. Table S7. Results of linear mixed models for picture rating task. Figure S5. Cumulative THC effect on picture rating. Table S8. Results of linear mixed models for cumulative THC effect on picture rating. Figure S6. Correlations between plasma cannabinoids and attentional bias. Figure S7. Correlations between plasma cannabinoids and picture rating. Figure S8. Study CONSORT flow diagram. [file ADD-119-322-s001.docx]
